# Phage-Displayed Mimotopes of SARS-CoV-2 Spike Protein Targeted to Authentic and Alternative Cellular Receptors

**DOI:** 10.3390/v14020384

**Published:** 2022-02-14

**Authors:** Valery A. Petrenko, James W. Gillespie, Laura Maria De Plano, Michael A. Shokhen

**Affiliations:** 1 Department of Pathobiology, College of Veterinary Medicine, Auburn University, Auburn, AL 36849, USA; 2 Department of Chemical, Biological, Pharmaceutical and Environmental Sciences, University of Messina, 98122 Messina, Italy; lauramaria.deplano@unime.it; 3 Department of Chemistry, Bar Ilan University, Ramat Gan 52900, Israel; michael.shokhen@biu.ac.il

**Keywords:** molecular mimicry, phage display, spike protein, SARS-CoV-2 virus, mimotope, virus receptors, alternative receptors, landscape phage

## Abstract

The evolution of the SARS-CoV-2 virus during the COVID-19 pandemic was accompanied by the emergence of new heavily mutated viral variants with increased infectivity and/or resistance to detection by the human immune system. To respond to the urgent need for advanced methods and materials to empower a better understanding of the mechanisms of virus’s adaptation to human host cells and to the immuno-resistant human population, we suggested using recombinant filamentous bacteriophages, displaying on their surface foreign peptides termed “mimotopes”, which mimic the structure of viral receptor-binding sites on the viral spike protein and can serve as molecular probes in the evaluation of molecular mechanisms of virus infectivity. In opposition to spike-binding antibodies that are commonly used in studying the interaction of the ACE2 receptor with SARS-CoV-2 variants in vitro, phage spike mimotopes targeted to other cellular receptors would allow discovery of their role in viral infection in vivo using cell culture, tissue, organs, or the whole organism. Phage mimotopes of the SARS-CoV-2 Spike S1 protein have been developed using a combination of phage display and molecular mimicry concepts, termed here “phage mimicry”, supported by bioinformatics methods. The key elements of the phage mimicry concept include: (1) preparation of a collection of p8-type (landscape) phages, which interact with authentic active receptors of live human cells, presumably mimicking the binding interactions of human coronaviruses such as SARS-CoV-2 and its variants; (2) discovery of closely related amino acid clusters with similar 3D structural motifs on the surface of natural ligands (FGF1 and NRP1), of the model receptor of interest FGFR and the S1 spike protein; and (3) an ELISA analysis of the interaction between candidate phage mimotopes with FGFR3 (a potential alternative receptor) in comparison with ACE2 (the authentic receptor).

## 1. Introduction

**Figure 1:**
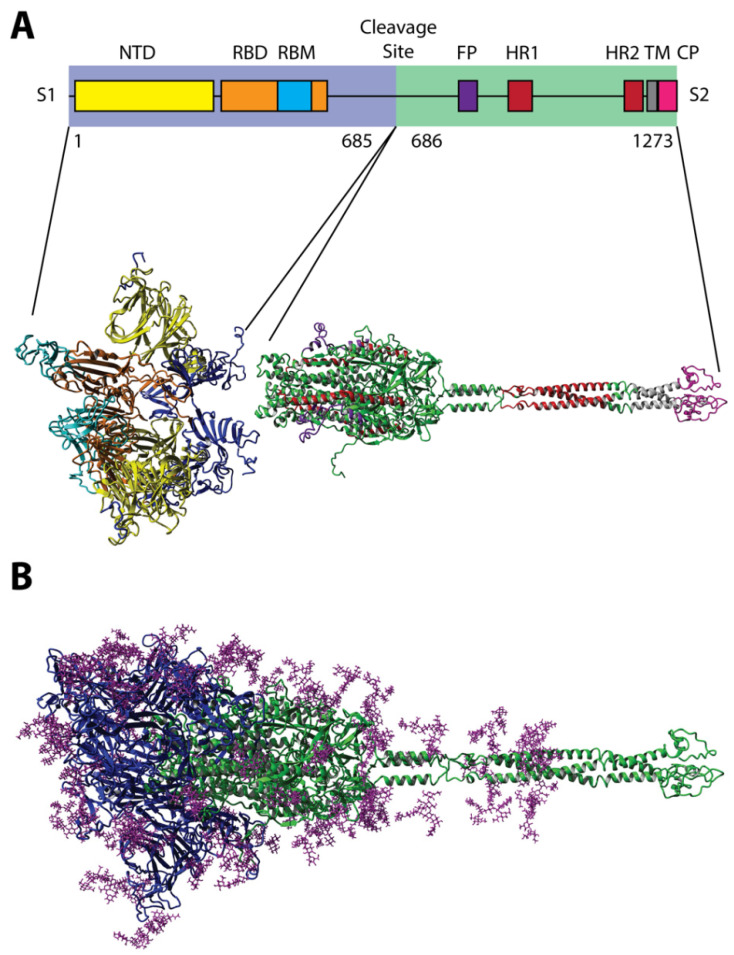
Three-dimensional predicted model of the spike (S) glycoprotein of the SARS-CoV-2 virus composed of (**A**) two well-defined structural domains (S1 and S2), decorated with (**B**) 22 N-glycan residues as modeled using 6VSB and 6VXX [38,39]. Monomers of the S protein, composed of polypeptide chains of 1273 amino acids, form homotrimer spikes on the virus surface [18]. Spike protein monomers are composed of three major structural domains: head, stalk, and cytoplasmic tail. The head comprises the N-terminal domain (NTD; yellow) and the receptor-binding domain (RBD; orange), which displays the receptor-binding motif (RBM; cyan) that is responsible for interaction with cell receptors [40]. RBDs in non-activated viral S glycoprotein trimers are present in a hidden “down” conformation. The S glycoprotein is cleaved by host proteases (trypsin and furin) at the site between the S1 and the S2 subunits [41,42]. The S2 domain of the S protein consists of fusion peptide (FP; purple), two heptad-repeat domains (HR1 and HR2; red), a transmembrane domain (TM; gray), and a cytoplasm domain (CP; pink). A second proteolytic site (S2′ site), located within the S2 subdomain, is also cut by type II transmembrane serine protease (TMPRSS2) as well as cathepsin B and L (CatB/L) to enable virus-cell fusion by triggering the dissociation of S1 and the irreversible refolding of S2, a conformational change of the S protein and the fusion of the viral envelope and endosomes [42].

**Figure 2:**
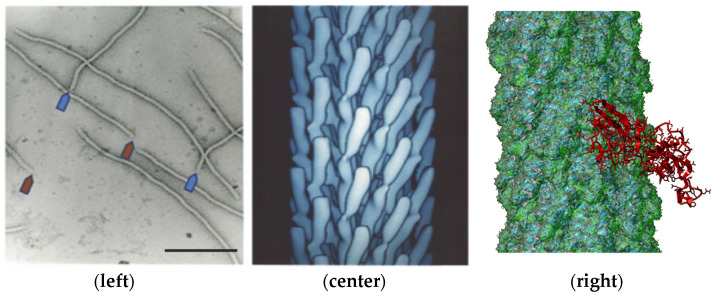
Electron microscopy image of filamentous phage (**left**) and electron density model (**center**) of filamentous phage M13 (courtesy of Lee Makowski and Gregory Kishchenko). Blue and red arrows depict the sharp and blunt ends of the phage capsid with attached minor coat proteins pIII/pIV and pVII/pIX, respectively (five copies each). Major coat protein (~2700 copies) forms the tubular capsid around viral single-stranded DNA (scale bar: 100 nm). 3D structure (**right**) of the complex between phage displaying the peptide EDYSELVSQ (green) with FGFR3 (red). Here, the peptide-displayed phages are designated by the structure of the inserted foreign peptides.

**Figure 3:**
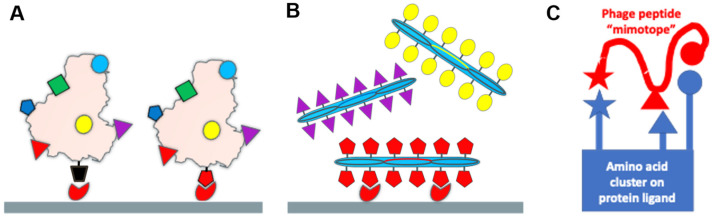
(**A**) Schematic of viral evolution (adaptation) leading to increased fitness to cellular receptors through diversification of viral functional domains; (**B**) schematic of SARS-CoV-2 functional domains and epitopes (or their mimetics) fused to p8 proteins and selected from landscape phage libraries through phage mimicry; (**C**) the phage-displayed peptide (mimotope) contains the same or similar amino acid (AA) residues as amino acid clusters (AA clusters) on the surface of spike protein, and presumably can interact with viral cellular receptors.

The evolution of the SARS-CoV-2 virus during the COVID-19 pandemic was accompanied by the emergence of a diverse assortment of new virus variants having multiple mutations in both receptor- and antibody-binding sites located throughout the spike S protein [1,2,3,4,5,6,7,8,9,10,11,12,13,14,15,16,17,18,19,20,21,22,23,24,25] (Figure 1). Analysis of a variety of coronavirus variants that caused epidemics during the last 60 years showed that their evolution may occur not only through the appearance and selection of immuno-resistant mutants, but also by the selection of variants with altered spike proteins that can use alternative, or “reserve”, human host receptors for entering into target cells [18,26,27,28,29]. Our current understanding of the molecular mechanisms of virus adaptation to human host cells [30] and immuno-resistant human populations is not sufficient to rationally design efficient countermeasures against viral pandemics, such as the SARS-CoV-2 virus. To empower increased insight into the evolutionary mechanisms of the SARS-CoV-2 spike protein, we suggest using recombinant filamentous bacteriophage-based probes [31], displaying on their surface an array of ~4000 foreign peptides, which produces a unique molecular landscape across the viral surface that can mimic the structure of viral receptor-binding sites [32,33,34,35,36,37], as illustrated in Figure 2 and Figure 3.

Phage display, a technique developed over the past 35 years, employs the use of engineered bacteriophages to display a genetically encoded peptide sequence fused onto an exposed structural protein of a bacteriophage particle, such as fd-tet [31]. Landscape phage libraries are composed of a collection of different bacteriophages or “phages” in which a fused protein is displayed on the N-terminus of the p8 major coat protein as opposed to the p3 minor coat protein, as seen with many commonly used phage display libraries [43,44]. Due to the structural modifications introduced by the displayed peptide fusions to the 4000 copies of the p8 protein, each landscape phage particle can be treated as a unique nanomaterial with novel and emergent properties that cannot be observed by use of an individual synthetic peptide alone [45]. In this p8-type phage display system, the array of densely packaged foreign peptides composes a unique landscape in which the structure and function of individual peptides can be influenced by interactions with neighboring peptides and the body of the phage particle [45,46]. For example, it was shown that synthetic peptides corresponding to phage-displayed peptides on landscape phages can demonstrate very different activity in competition ELISA. This property of landscape phages demonstrates the potential of each phage to be a selectable nanomaterial, as opposed to simply a means to discover binding peptides. The discovery of landscape phage mimotopes specific towards viral attachment proteins, such as the SARS-CoV-2 spike protein, provides a unique nanomaterial that can be translated to various applications, such as vaccine products, biosensors, or other analytical devices [44].

Molecular or antigenic mimicry can be used to describe the similarity of different antigens that can cause the cross-recognition of conformational peptides/proteins or amino acid sequence to a receptor, commonly observed between pathogens and immune receptors that triggers an undesired autoimmune response [47,48]. We extend this concept and use in silico computational tools previously developed for studying phage-displayed peptide sequences discovered between antibody-antigen interactions [49].

In this study, we sought to discover phage mimotopes of the SARS-CoV-2 spike S protein using the concepts of phage display and molecular mimicry [31], termed here “phage mimicry.” Three essential steps in the phage mimicry paradigm can be defined: (1) enrichment of a multibillion clone population of landscape phage libraries for thousands of phage variants that interact with cellular receptors exposed on the surface of mammalian normal and cancer cells; (2) identification of computationally predicted amino acid clusters with similar 3D conformations as naturally occurring ligands of the target receptor; and (3) evaluation of candidate phage mimotopes for their ability to interact with a receptor of interest in vitro or in vivo using an ELISA and other diagnostic methods in comparison with phages displaying a non-related peptide.

Here, we used a collection of landscape phages that were previously selected by our research group through enrichment of landscape phage display libraries for cancer cell binding/penetrating phages using a panel of human cancer cell lines, which overexpress a variety of different receptors, including receptors that presumably can be involved in viral pathogenesis. Using the PepSurf algorithm, which maps a set of affinity-selected peptides onto the surface of a solved PDB structure, the linear peptide sequence that mimics the genuine epitope (mimotopes) of the antibody-antigen complex can be identified. The discovered linear peptide sequences can therefore mimic the complex 3D conformational folding of the native ligands. We hypothesized that instead of using peptide sequences discovered against an antibody to discover the molecular mimotopes of the antigen, as was presented in the original manuscript, we could use a library of preselected phages enriched towards cellular receptors to discover viral protein molecular mimotopes involved with viral attachment. Next, we studied the interaction of phage mimotopes mimicking amino acid clusters in the SARS-CoV-2 S1 protein that bind the canonical receptor ACE2 as a proof-of-concept model. We identified amino acid clusters on two different ligands (SARS-CoV-2 S1 protein, FGF1, and NRP1), which presumably interact with the FGFR3 receptor. We predicted conformationally similar domains of phage mimotopes interacting with the same cellular receptor FGFR. Finally, we analyzed candidate phage mimotopes for binding with recombinant FGFR3.

## 2. Materials and Methods

A panel of phage mimotopes of the SARS-CoV-2 spike S protein that bind certain cell receptors was developed using the phage mimicry technique [31,50,51], supported by a combination of bioinformatic methods [49,50,51,52] and validated by experimental methods.

### 2.1. Preparation of Libraries of Phages That Display Receptor-Binding Mimotopes Fused to the Major Coat Protein p8

The source of SARS-CoV-2 mimotopes was the p8-type polyvalent peptide phage-displayed library (or, shortly, the landscape phage library) [43,44,53]. Bacteriophage fd (Figure 2), which itself has no natural tropism to mammalian cells, is a suitable vector for generating random peptide phage-displayed libraries—a rich source of ligands for cellular receptors. It was shown that phages selected from these libraries can specifically recognize cellular receptors and penetrate into sub-cellular compartments during their artificial molecular evolution in vitro and in vivo, similarly to evolution observed with naturally evolved viruses. We demonstrated that selection of organ/cell-associated phage variants from their multibillion clone libraries and bioinformatic analysis of their cell-binding peptides in comparison with proteins of natural viruses allow the identification of functional virus-host binding sites that are apparently used during viral pathogenesis. The phages in these libraries have been screened against a variety of human cancer and normal cells harboring cellular proteins in their native structure-functional state [54]. Specifically, in this project we used an assortment of several hundred phage clones isolated from human lung [55], prostate [53], pancreatic [56], and breast cancer [57] cells and characterized for specificity and selectivity towards corresponding cell lines.

### 2.2. Mapping of Epitopes and Receptor-Binding Sites on the Surface of Spike S Protein

From a variety of computer programs that were prepared for studying antibody-antigen interactions, we chose PepSurf [49], which originally was designed for mapping a set of affinity-selected peptides onto the resolved structure of the antigen, and was adapted in this work for the discovery of receptor-binding mimotopes of SARS-CoV-2 S protein. The important feature of this algorithm is that it assumes that the peptides mimic surface residues (i.e., solvent exposed residues), and buried residues are eliminated from the search. In this application, the S protein played a role of “antigen” and mimotopes interacting with virus receptors were identified by aligning them against all possible paths in the graph and clustering the most significant matches in which a predicted epitope and/or receptor-binding site is implied. To discover phage-displayed mimotopes of epitopes and receptor-binding clusters on the surface of the spike protein, the PepSurf server was used, as recommended for epitope mapping, with the standard input of the Protein Data Bank (PDB) files of the S protein with a solved 3D structure available from the PDB (6VYB and 6M0J) and a set of peptides discovered by screening of landscape phage-displayed libraries against human cancer cells in vitro and in vivo. The first output of the program is the alignment of each peptide to the 3D structure of the spike. When several peptides were aligned, the server implemented a clustering algorithm to detect one or more patches of residues on the surface of the surveyed protein. Thus, the second output is the predicted patches. Such a patch may correspond to a putative epitope site on the spike, or as assumed in our work, a receptor-binding site.

### 2.3. Molecular Modeling

The 3D structure of a landscape phage displaying the peptide EDYSELVSQ as an N-terminal fusion to all copies of the mature p8 major coat protein was generated by SWISS-MODEL, the homology modeling server [58,59,60,61,62]. The full 55 amino acid residue sequence of p8 (AEDYSELVSQPAKAAFDSLQASATEYIGYAWAMVVVIVGATIGIKLFKKFTSKAS) was used as the input query, where the residues highlighted in bold indicate the displayed N-terminal peptide fusion on every copy of p8. The 3D structure of 1FDM, from the PDB, was identified as a template by a search performed against the SWISS-MODEL template library with BLAST [63] and HHBlits [64]. The resulting 3D structure was a fragment formed by 55 coat proteins of filamentous phage, where the full-length of the latter is ~2700 copies of the coat proteins. It should be noted that the model generated by homology modeling coat proteins had a reduced sequence containing 6–55 residues only from the target model. All 55 reduced-coat proteins were substituted for the full-length intact variant by means of molecular modeling procedures implemented in the YASARA Structure software package [65,66]. Finally, the 3D structure of the phage fragment with corrected protein sequences was optimized under an AMBER ff14SB force field [67] in a periodic solvent box with explicit water molecules and physiological concentrations of Na^+^ and Cl^−^ ions.

The 3D structures of the complexes formed by the fibroblast growth factor receptor 3 (FGFR3) with ligands phage, FGF1, NRP1, and SARS-CoV-2 spike RBD, presented in Figure 8, were predicted by the HADDOCK web server for protein-protein docking [68,69]. The 3D structures of receptor and ligands used as input data for the docking procedure were acquired from the following crystal structures available from the PDB: FGFR3 (1RY7), FGF1 (1RY7), NRP1 (2QQN), and the SARS-CoV-2 spike protein RBD (6M0J).

### 2.4. Antibodies and Human Recombinant Proteins

The following commercially available antibodies were used in this study: rabbit; the anti-SARS-CoV-2 spike protein mAb (Sino Biological, Wayne, PA, USA #40150-R007, RRID: AB_2827979); and HRP-conjugated goat anti-rabbit IgG (H+L) polyclonal antibody (Jackson ImmunoResearch, West Grove, PA, USA #111-035-045, RRID: AB_2337938).

The following human recombinant proteins were obtained from Sino Biological, Wayne, PA, USA (Table 1): ACE2-His (#10108-H08H), DPP4-His (#10688-H08H), FGFR3-His (#16044-H08H), and the SARS-CoV-2 S1-His spike protein (40591-V08H). All recombinant proteins used in this study were resuspended to a stock concentration of 250 µg/mL in ddH_2_O, according to the manufacturer’s instructions.

### 2.5. Phage Indirect ELISA with Human Extracellular Receptors

An indirect ELISA was used to evaluate the interaction between landscape phages displaying recombinant peptide fusions of SARS-CoV-2 spike protein mimotopes and candidate host cellular receptors. Recombinant, extracellular domains of human receptors (hACE2, hNRP1, and hFGFR3) were diluted to a final concentration of 2.0 µg /mL with a coating buffer (1× PBS, pH 7.4). Wells of a 96-well, high-binding microplate (Corning, Corning, NY, USA #9018) were coated with 100 µL of each diluted candidate receptor (200 ng/well) and incubated overnight at 4 °C.

Unbound antigen was removed, and the wells were washed three times with 200 µL of washing buffer (1× PBS, pH 7.4/0.1% Tween 20) at room temperature. Antigen-coated wells were blocked with 200 µL of blocking buffer (1× PBS, pH 7.4/2% BSA/0.1% Tween 20) for 1 h at 37 °C. The wells were then washed three times with 200 µL of washing buffer at room temperature. Candidate phages were diluted to a working concentration of 5.0 × 10^11^ vir/mL and tenfold serial dilutions were prepared to a final concentration of 5.0 × 10^5^ vir/mL in blocking buffer. The wells were then treated with 100 µL of diluted phages for 1 h at 37 °C. The wells were washed four times with 200 µL of washing buffer at room temperature. Bound phages were detected using 100 µL of rabbit, anti-fd phage IgG [70,71] diluted 1/1000 in blocking buffer for 1 h at 37 °C. The wells were washed four times with 200 µL of washing buffer at room temperature. Bound rabbit IgGs were detected using 100 µL of horseradish peroxidase (HRP)-conjugated goat anti-rabbit secondary antibody diluted 1/40,000 in blocking buffer for 1 h at 37 °C. The wells were washed four times with 200 µL of washing buffer prior to adding 50 µL of 1-Step Ultra TMB-ELISA Substrate (ThermoScientific, Waltham, MA, USA). The reduction of 3,3′,5,5′-tetramethylbenzidine (TMB) substrate by the HRP-conjugated antibody was monitored over a 30 min incubation at room temperature. The reaction was stopped by adding 50 µL of Stop Solution (0.2 M sulfuric acid) to each well. Endpoint absorbances for each well were measured at 450 nm using a Synergy H1 plate reader (BioTek, Winooski, VT, USA).

### 2.6. Indirect ELISA with Candidate SARS-CoV-2 Spike Protein Receptors

An indirect ELISA was used to evaluate the interaction between a recombinant SARS-CoV-2 spike protein and candidate host cellular receptors. Recombinant, extracellular domains of candidate SARS-CoV-2 S1 receptors (hACE2, hDPP4, hNRP1, and hFGFR3) were diluted to a final concentration of 2.0 µg/mL with a coating buffer (1× PBS, pH 7.4). Bovine serum albumin (BSA), IgG-free and protease-free, (Jackson ImmunoResearch, West Grove, PA, USA #001-000-173, RRID: AB_2336947) was diluted to a final concentration of 2% with the coating buffer and used as a negative control sample. Wells of a 96-well, high-binding microplate (Corning, Corning, NY, USA #9018) were coated with 100 µL of each diluted candidate receptor (200 ng/well) in triplicate and incubated overnight at 4 °C. A set of wells was treated with the coating buffer as a control for plastic binding.

Unbound antigen was removed, and the wells were washed three times with 200 µL of washing buffer (1× PBS, pH 7.4/0.1% Tween 20) at room temperature. Antigen-coated wells were blocked with 200 µL of blocking buffer (1× PBS, pH 7.4/2% BSA/0.1% Tween 20) for 1 h at 37 °C. The wells were then washed three times with 200 µL of washing buffer at room temperature. The recombinant SARS-CoV-2 S1-His spike protein was diluted to a final concentration of 2.0 µg/mL with blocking buffer. The wells were then treated with 100 µL of diluted SARS-CoV-2 S1 spike protein (200 ng/well) for 1 h at 37 °C. The wells were washed four times with 200 µL of washing buffer at room temperature. Bound SARS-CoV-2 spike protein was detected using 100 µL of the rabbit anti-SARS-CoV-2 spike protein monoclonal antibody diluted 1/5000 in blocking buffer for 1 h at 37 °C. The wells were washed four times with 200 µL of washing buffer at room temperature. Bound rabbit IgGs were detected using 100 µL of horseradish peroxidase (HRP)-conjugated goat anti-rabbit secondary antibody diluted 1/80,000 in blocking buffer for 1 h at 37 °C. The wells were washed four times with 200 µL of washing buffer prior to adding 50 µL of 1-Step Ultra TMB-ELISA Substrate (ThermoScientific, Waltham, MA, USA). The reduction of 3,3′,5,5′-tetramethylbenzidine (TMB) substrate by the HRP-conjugated antibody was monitored over a 30 min incubation at room temperature. The reaction was stopped by adding 50 µL of Stop Solution (0.2 M sulfuric acid) to each well. Endpoint absorbances for each well were measured at 450 nm using a Synergy H1 plate reader (BioTek, Winooski, VT, USA).

### 2.7. Data and Statistical Analysis

Descriptive statistics for endpoint values were calculated for each sample. Due to the small sample size, nonparametric statistical tests were selected to analyze the differences in the data. A Kruskal-Wallis one-way analysis of variance (ANOVA) was performed using R (version 4.1.1) to identify whether there was a statistical difference in mean endpoint absorbances between candidate receptors. A post-hoc Dunnett’s test for multiple comparisons was used to identify statistically significant differences in mean endpoint absorbances when compared to a negative control protein (BSA).

## 3. Results

### 3.1. Landscape Phage Libraries

To identify landscape phage mimotopes of the SARS-CoV-2 spike protein that bind a certain cellular receptor (Figure 2) [44], phage collections were prepared by screening the multibillion parental phage libraries against a variety of human cancer cells expressing authentic functional receptors: human lung [55], prostate [53], pancreatic [56], and breast cancer [57] cells, some of which, along with other human cancer cells, have been used for the propagation of SARS-CoV and SARS-CoV-2 in cell culture [30,72,73,74,75,76,77]. We assumed that after treating target cells with the phage library and discarding unbound phages, a number of phage mimics of the SARS-CoV-2 virus remained bound to cellular receptors; for example, previously identified nucleolin [57], integrins [73], and proteinase N [73], as illustrated in Figure 3B. To discover phage-displayed mimotopes that mimic the receptor-binding AA cluster on the surface of the SARS-CoV-2 spike protein (Figure 3C), we used the PepSurf server as recommended for epitope mapping, as discussed above (Section 2.2). We evaluated the performance of phage mimicry using representatives of major classes of cell surface receptors, which mediate entry of coronaviruses into the host cells: (1) metallopeptidases, including angiotensin-converting enzyme 2 (ACE2) [1,26], and (2) growth factor receptors, including fibroblast growth factor receptor 3 (FGFR3) [35,37,78,79,80,81].

### 3.2. Identification of Receptor-Binding Domain Mimotopes on the SARS-CoV-2 Spike Protein

In the traditional phage-display applications, such as epitope discovery and vaccine development, the final goal is discovery of peptides that can be used as a replacement for natural protein antigens [31]. However, in most proteins, including the spike protein, the epitopes and receptor-binding sites are presented by conformational AA clusters, in which separated AAs are brought together through a specific folding of proteins. That is why the goal of our project was the development of phage mimotopes, which can be used themselves as counterparts or artificial ligands of cellular receptors and antibodies in different applications, including the study of viral evolution and the design of diagnostic systems and elements of molecular vaccines [44,82].

**Figure 4:**
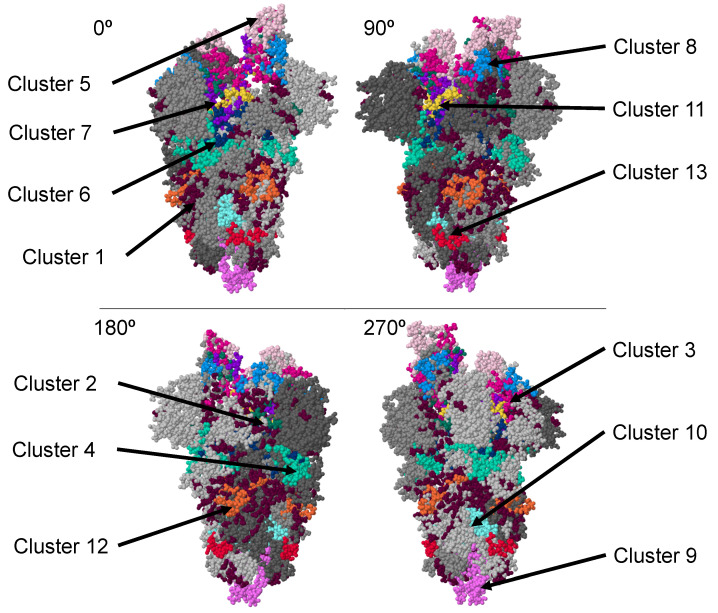
Clusters of amino acids identified by PepSurf surface accessible alignments of cell receptor-binding phage mimotopes on the SARS-CoV-2 spike protein using the PDB model 6VYB. Cluster 1 (dark purple), cluster 2 (green), cluster 3 (bright pink), cluster 4 (turquoise), cluster 5 (pink), cluster 6 (dark blue), cluster 7 (purple), cluster 8 (blue), cluster 9 (dark pink), cluster 10 (light blue), cluster 11 (yellow), cluster 12 (orange), and cluster 13 (red).

To identify receptor-binding domain mimotopes located on the SARS-CoV-2 spike protein, we collected a panel of over 350 phage displayed peptides that we previously enriched and characterized for binding to various human cancer cell lines [53,55,56,57]. As many receptors and growth factors relevant for increased cell proliferation are overexpressed in cancer cell lines, we hypothesized that the phage-display libraries enriched for binders to these various cancer cell types would provide a representative panel of relevant phages displaying receptor-binding peptides for use in this study. We first analyzed various sets of these cell receptor-binding peptides to generate an alignment cluster of amino acids on the surface of the full-length SARS-CoV-2 spike protein based on the 3D PDB model 6VYB. Over 100 different alignment clusters were identified on the surface of the SARS-CoV-2 spike protein using the PepSurf alignment algorithm, which defines a network of surface-accessible residue paths and aligns peptide inputs to these residue paths. Alignment paths or AA clusters that contain several peptides were scored higher and were hypothesized to be more functional. After an initial round of peptide enrichment to identify SARS-CoV-2 spike protein mimics, we performed another round of mimotope screening, in which individual peptides were used as input into the PepSurf program to identify the best surface alignment of each peptide (AA clusters) on the 6VYB 3D spike model. We then performed a hierarchical clustering of peptides with the 6VYB model of the SARS-CoV-2 spike protein (Figure 4).

**Figure 5:**
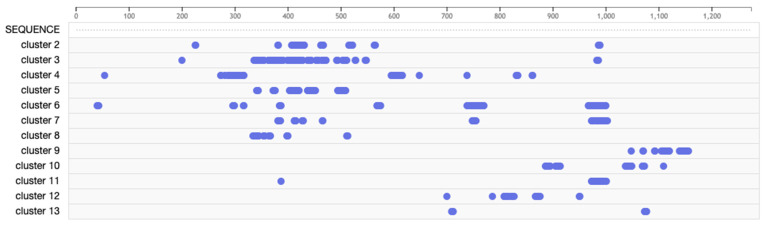
Amino acid residues corresponding to each identified functional cluster.

Amino acid residues were grouped into 13 potentially functional domains (Figure 5). Cluster 1 appeared to contain amino acids that did not strongly cluster into any functional domain. We speculate that these amino acid residues are the result of non-specific binding of phages in the parental library enrichment procedures. The remaining 12 amino acid clusters appear to contain more residues that cluster into common domains. Some of these clusters overlap (clusters 2, 3, and 5–8) but involve additional residues that may only be functional during specific orientations/conformations of the spike protein. For example, as the spike protein transitions from the closed to open state, additional functional amino acids are exposed, but also previously functional residues may no longer be functional based on their accessibility [83]. However, several of the identified clusters define isolated regions within the spike protein, including clusters 9, 4, and 10. Some of the clusters, i.e., cluster 4, contain residues that are specific for that cluster but cover a wide distribution of positions that do not cluster around a central region. These regions most likely contain functional domains that are highly active or functionally conserved.

Following identification of spike protein functional domains using our bioinformatics approach, we observed that most of the functional amino acid residues were in the spike receptor-binding domain (RBD) as expected and chose to focus our attention on interactions located within this known functional region using the PDB model 6M0J.

### 3.3. Phage-Displayed Mimotopes of Spike S1 Protein Interacting with ACE2

**Figure 6:**
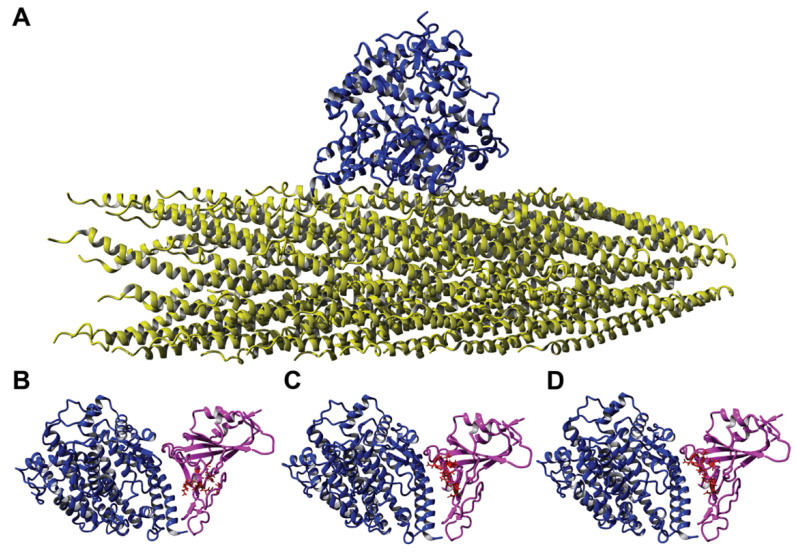
Interaction of ACE2 with different ligands. (**A**) Interaction of a landscape phage displaying the peptide DGRADLSYD on the full-length p8 protein (yellow) with ACE2 (blue) as determined using homology modeling. Here, a segment containing less than 1% of the landscape phage is presented, where the DGRADLSYD peptide is presented as an N-terminal fusion to all copies of the mature p8 major coat protein. Molecular model 6M0J demonstrating the interaction between ACE2 protein (blue) and recombinant SARS-CoV-2 spike RBD (pink) with amino acid clusters corresponding to phage mimotopes. (**B**) DGRADLSYD; (**C**) VGIDEQRAD; and (**D**) DGRSIVGDE, highlighted in red.

To validate the phage mimicry approach for the discovery of phage mimotopes that correspond to functionally active sites on the surface of the SARS-CoV-2 spike protein, we focused on the SARS-CoV-2 spike protein RBD. We hypothesized that phage-displayed peptide mimotopes would share common amino acid clusters with the ACE2-interacting S protein’s AA clusters as visualized through the PDB model 6M0J [84]. We narrowed our collection of spike protein mimics to those that contained residues involved in mediating the spike-ACE2 interaction and visually confirming a potential interaction between the two proteins in YASARA. Using these criteria, we narrowed the pool of potential candidates to 55 phages. We hypothesized that peptides containing the most amino acid residues in common with the spike RBD would have highest interaction potential and therefore produce the highest binding in a functional ELISA. The peptides were then ranked based on the number of amino acids shared with the SARS-CoV-2 spike RBD, resulting in a panel of 11 phages that were used for screening in a functional ELISA towards a recombinant ACE2 protein. For example, the phage-displayed peptides DGRADLSYD, VGIDEQRAD, and DGRSIVGDE all contained 9 amino acid residues in common with the spike RBD and were located in the spike-ACE2 interaction site (Figure 6B–D). We then modeled the interaction of a landscape phage displaying the DGRADLSYD peptide on all copies of the p8 major coat protein with ACE2 to demonstrate the various sites of interaction between the phage-ACE2 complex, as illustrated in Figure 6A.

**Figure 7:**
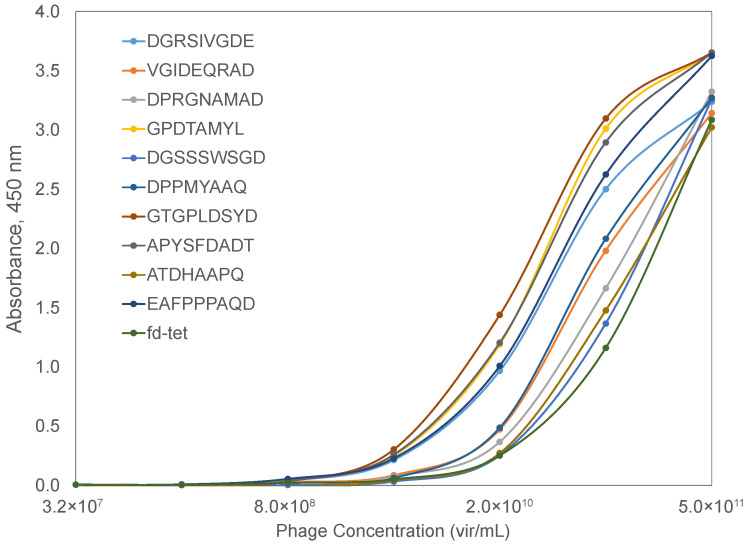
ACE2-binding phages displaying SARS-CoV-2 spike RBD-mimotopes as characterized by an indirect ELISA. Serial dilutions of phages were incubated with a bound ACE2 receptor, followed by incubation with a rabbit anti-phage IgG and an HRP-conjugated goat anti-rabbit IgG. Signal was produced using a TMB substrate and endpoint absorbance at 450 nm was measured after a 30-min incubation. Candidate RBD-binding phages were compared to the wildtype fd-tet phage (dark green).

To check the prediction value of the phage mimicry strategy, we characterized the binding of phages displaying the SARS-CoV-2 spike protein RBD-mimicking peptides to a recombinant ACE2 protein in a functional ELISA. Briefly, a recombinant ACE2 protein was adsorbed to the wells of a 96-well plate and blocked with BSA. Serial dilutions of candidate phages were prepared and allowed to interact with bound ACE2 at 37 °C for 1 h. Following extensive washing, bound phages were detected using a rabbit anti-phage antibody and a goat anti-rabbit secondary antibody conjugated with horseradish peroxidase. The signal was generated using a TMB substrate and absorbance data collected over a 30 min interval to calculate the maximum velocity for each reaction well. Spike RBD-mimicking phages produced higher binding in an ELISA when compared to the unrelated parent phage, fd-tet, suggesting that the identified mimotopes serve as functional peptide mimics of the SARS-CoV-2 spike protein (Figure 7). Based on the predicted interaction location provided by the molecular modeling of AA clusters of the SARS-CoV-2 spike RBD and ACE2, the affinity of the phage mimotope to the ACE2 receptor can be modified based on differences in the amino acids involved in the interaction site.

### 3.4. Identification of Phage-Displayed Mimotopes of the Spike S1 Protein Interacting with FGFR3

Since there is no data in the literature concerning 3D molecular models demonstrating the hypothetical complex between FGFR and the SARS-CoV-2 spike protein that are confirmed by X-ray crystallography or electron microscopy analysis, as in the model described above for the ACE2 and SARS-CoV-2 spike protein complex, we used for our analysis the phage mimicry strategy. The phage mimicry strategy is based on principles of equivalence relations. In mathematics, if object A is equivalent to object B, and object B is equivalent to object C, then object A is also equivalent to object C, according to the transitivity relationship. In virology, molecular mimicry can be defined as a structural similarity between viral proteins and natural ligands of human cellular receptors. In relation to the discovery of phage mimotopes, which are able to compete with RBD for binding to FGFR3, we assumed that they can be found among FGFR-binding phage mimotopes of the natural ligands for FGFR3—neuropilin-1 (NRP1) and fibroblast growth factor 1 (FGF1), which form stable complexes with FGFR3 [81,85,86,87,88]. Since the 3D structure of the complex between NRP1 and FGFR3 is unknown, we used methods of molecular modeling. We found that the CUB2 domain of NRP1 has amino acid clusters with similarity to the SARS-CoV-2 spike RBD and phage mimotopes.

**Figure 8:**
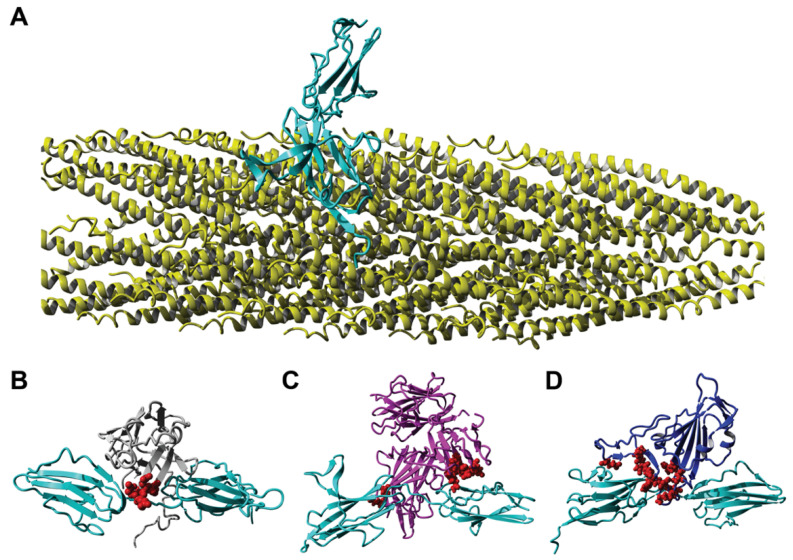
Interaction of fibroblast growth factor receptor 3 (FGFR3) with different ligands: (**A**) Interaction of a landscape phage displaying the peptide EDYSELVSQ (yellow) on the full-length p8 protein with FGFR3 (cyan), as determined using homology modeling. Here, a segment containing less than 1% of the landscape phage is presented, where the EDYSELVSQ peptide is presented as an N-terminal fusion to all copies of the mature p8 major coat protein. Interaction of FGFR3 (cyan) with (**B**) FGF1 (gray), (**C**) NRP1 (magenta), or (**D**) SARS-CoV-2 spike RBD (blue), with amino acid clusters containing alignments to the EDYSELVSQ mimotope highlighted in red.

In a similar manner, we determined the structure of the complex between FGFR3 and the SARS-CoV-2 spike protein RBD (Figure 8). The assortment of FGFR3 phage binders was extended using the model of the complex between FGFR3 and FGF1. Mimotopes were identified using the alignment of phage peptides with the FGF1-FGFR3 complex (PDB model 1RY7). Using the same selection criteria as above, we narrowed the pool of candidate phages displaying FGFR3-binding mimotopes to 13 peptide sequences. We prepared PepSurf surface residue alignments of each candidate mimotope to three different ligands, FGF1, NRP1, and the SARS-CoV-2 spike protein RBD (Table 2). From this analysis, we identified amino acid residues that were conserved between the phage-displayed mimotope and the amino acid clusters located on the three ligands. We observed that the identified mimotopes shared several amino acid residues in common with the three ligands. For example, the mimotope DGRMTVYNE contained a shared DGR motif that was common among all three ligands. Other residues in the mimotope were varied in different positions, based on the PepSurf alignment. However, we found that all amino acid clusters identified on the different ligands shared at least five amino acid residues in common, resulting in FGFR3 putative binding mimotopes.

**Figure 9:**
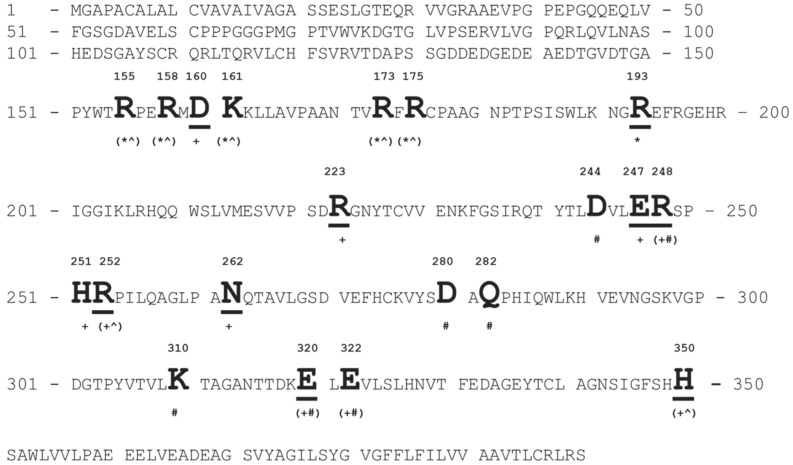
Primary structure of the FGFR3 domain. Amino acids involved in the interaction (designated by capital bold letters) between FGF1 (*), NRP1 (^), and RBD (#) were identified using the YASARA Structure and literature data. Promiscuous amino acid residues of FGFR3 involved with the interaction of FGF1, NRP1, and spike RBD are marked with (*^#). Members of AA clusters corresponding to the phage mimotope EDYSELVSQ are marked with (+) and indicated by underlined letters.

Using the primary protein sequence of the FGFR3 extracellular domain, we identified critical residues that mediated interactions between the receptors and the three ligands (Figure 9). We observed that several critical amino acid clusters around positions 155–175, 244–262, and 310–322 were important for the generation of the FGFR3-binding mimotope. We highlighted the amino acid residues of the phage mimotope EDYSELVSQ, which interact with FGFR3 (Figure 8A), and found that many of these residues, for example D160, E247, R248, and E320, were shared among the three ligands, which can confirm the localization of the ligand-binding site on the surface of FGFR.

### 3.5. Analysis of Interactions of Candidate Phage Mimotopes with FGFR3

**Figure 10:**
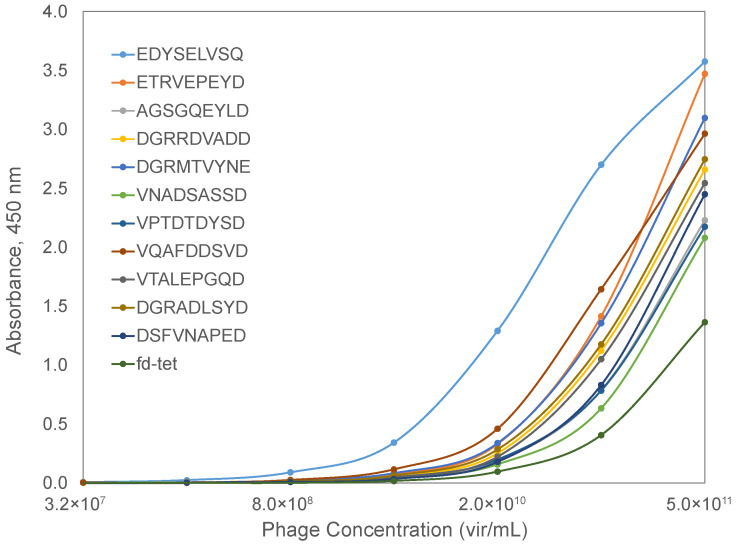
FGFR3-binding phages displaying FGF1 mimotopes as characterized by an indirect ELISA. Serial dilutions of phages were incubated with the bound FGFR3 receptor, followed by incubation with a rabbit anti-phage IgG and an HRP-conjugated goat anti-rabbit IgG. Signal was produced using a TMB substrate and endpoint absorbance at 450 nm was measured after a 30-min incubation. Candidate FGFR3-binding phages were compared to the wildtype fd-tet phage (dark green).

To test the functional activity of the identified FGFR3-binding phage mimotopes, we evaluated them in an indirect ELISA assay to verify binding to the extracellular domain of the FGF3 receptor. As above, the recombinant FGFR3 protein, which contained all of the extracellular domains of the receptor, was adsorbed to the wells of a 96-well plate and blocked with BSA. Serial dilutions of candidate phages were prepared and allowed to interact with bound FGFR3 at 37 °C for 1 h. Phages were detected using a rabbit anti-phage antibody and a goat anti-rabbit secondary antibody conjugated with horseradish peroxidase, as above. FGFR3-mimicking phages produced higher binding in the indirect ELISA, when compared to the unrelated parent phage, fd-tet, suggesting that the identified mimotopes serve as functional peptide mimics of the FGFR3 cellular receptor (Figure 10). Of the identified mimotopes, the phage-displaying peptides EDYSELVSQ and ETRVEPEYD demonstrated the highest binding activity in the FGFR3-binding assay. These peptides had stronger similarities to the NRP1 and spike RBD ligands than the natural FGF1 ligand we identified from the PepSurf alignment to each PDB structure, suggesting that these peptides would mimic the binding for all three proposed ligands.

### 3.6. Evaluation of Candidate SARS-CoV-2 Spike Protein Receptors

**Figure 11:**
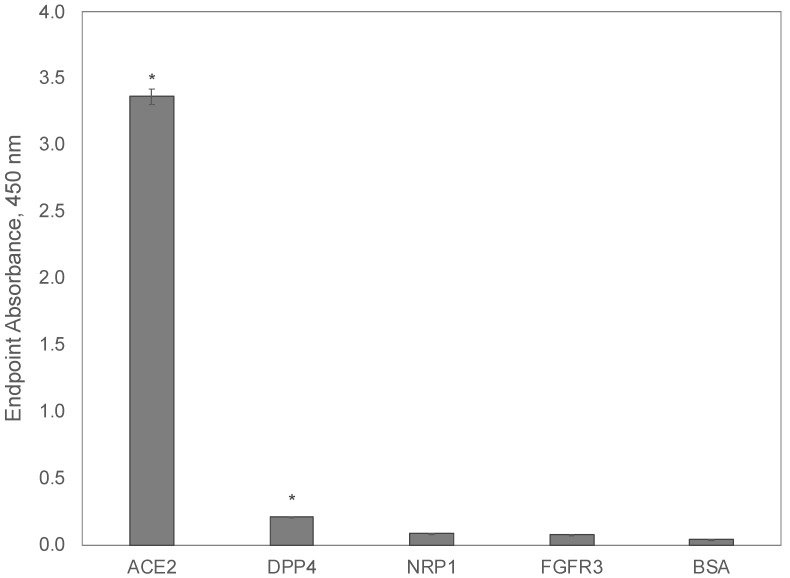
Evaluation of receptors interacting with the SARS-CoV-2 S1 spike protein by an indirect ELISA. Recombinant SARS-CoV-2 S1-His protein was incubated with bound ACE2, DPP4, FGFR3, NRP1, or BSA protein in triplicate wells for 1 h at 37 °C. The wells were incubated with a rabbit anti-SARS-CoV-2 spike IgG and an HRP-conjugated goat anti-rabbit IgG for 1 h at 37 °C. Signal was produced using a TMB substrate and endpoint absorbance at 450 nm was measured after a 30-min incubation. Mean endpoint absorbances for recombinant protein receptors were compared to the means of an unrelated BSA control using a Dunnett’s test with statistically significant differences (*p* < 0.05) indicated with a star.

We hypothesized that the SARS-CoV-2 S1 spike protein can interact with several host receptors and serves as a reservoir for additional mutations, which enables increased infection through the use of alternative host cell receptors. Here, we evaluated the binding of a recombinant SARS-CoV-2 S1 spike protein to interact with the extracellular domain of the ACE2 receptor [84], in comparison to the dipeptidyl peptidase 4 (DPP4/CD26) receptor identified as the primary receptor involved in MERS-CoV [89], neuropilin-1 (NRP1), a SARS-CoV-2 co-receptor that may facilitate improved viral entry into host cells [90,91,92], and fibroblast growth factor receptor 3 (FGFR3/CD333) [35]. Recombinant extracellular domains of candidate host receptors were adsorbed to a 96-well microplate before the addition of a recombinant SARS-CoV-2 S1 spike protein, including the receptor-binding domain (319–541), an integrin-binding motif (403–405), and an ACE2 receptor-binding domain (437–508). The bound S1 spike protein was determined using an indirect ELISA with an anti-SARS-CoV-2 spike protein antibody and an appropriate HRP-conjugated secondary. After measurement of endpoint absorbances for each sample, a Kruskal-Wallis one-way ANOVA was performed to identify differences in endpoint means across all samples (*p* = 8.88 × 10^−3^). A post-hoc Dunnett’s test was then performed against each candidate receptor to identify statistically significant differences in the average endpoint absorbance among samples, compared to a control (BSA), as summarized in (Figure 11).

As expected, the SARS-CoV-2 S1 spike protein domain demonstrated the highest binding to the canonical receptor ACE2, which contained the single extracellular domain of the host receptor, including a previously identified interaction site with the SARS-CoV-2 spike glycoprotein (30–41, 82–84, 353–357), a substrate-binding domain (345–346), an ADAM17 cleavage site (652–659), and a TMPRSS11D/TMPRSS2 cleavage site (697–716). As opposed to previous reports [89] that predicted that the SARS-CoV-2 S1 protein would have a high affinity to the DPP4 receptor from in silico methods, we demonstrated that binding is higher towards the ACE2 receptor. However, it is speculated that portions of the DPP4 binding site may still be present within the S1 protein of SARS-CoV-2 variants leaving the possibility for a receptor reversion back to using DPP4 as the primary receptor, given the appropriate selective pressure. Finally, we tested the binding of the SARS-CoV-2 S1 protein to both the NRP1 and FGFR3 extracellular domains as an example of an alternative receptor that could be used to broaden the tissue tropism of host-binding or to increase the infectivity/propagation of viral variants. In contrast to the synthetic S1 spike protein, phage mimotopes demonstrated strong specific binding to both ACE2 and FGFR3, while the S1 fragment of the viral spike protein demonstrated strong binding to ACE2 and DPP4 and only background binding to NRP1 and FGFR3. These results can demonstrate the insignificant role of FGFR3 alone as a receptor interacting with the SARS-CoV-2 spike protein, in comparison with ACE2, DPP4, and other alternative receptors of human coronaviruses. However, these findings leave a prospect for proposing the role of FGFR as a co-receptor that enhances the activity of ACE2, in a similar mechanism that was revealed for NRP1, in which overexpression enhances the infectivity of the SARS-CoV-2 virus, although the interaction of the spike RBD-NRP1 was not revealed [92,93,94,95,96]. To study this opportunity, phage mimotopes derived from different spike protein variants can be further studied in live human cell lines. In opposition to the spike-binding antibodies commonly used in studying the interaction of the ACE2 receptor with SARS-CoV-2 variants in vitro, phage spike mimotopes targeted to other cellular receptors would allow the discovery of their role in viral infection in vivo using cell culture, tissue samples, or whole organisms. For example, in novel, emerged variants of SARS-CoV-2, including the recent Omicron strain, different configurations of the spike RBD and the role of alternative receptors can be revealed using more advanced methods for studying viral-host interactions and using phage mimotopes prepared using the phage mimicry strategy presented in this study. The higher performance of phage mimotopes can be explained by the multivalency of landscape phage probes and the involvement of amino acid residues in neighboring p8 subunits of the phage in the target receptor-binding. This remarkable characteristic of the landscape phage can be used in the design of molecular devices to control virus-host interactions.

### 3.7. Primary Structure Analysis of Receptor-Binding AA Clusters of SARS-CoV-2

**Figure 12:**
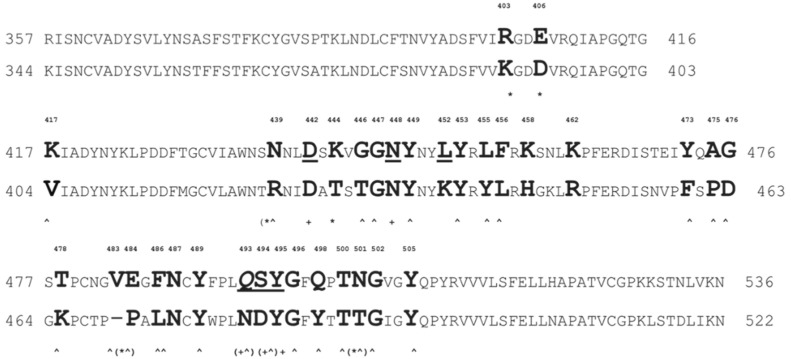
Primary protein structures of the RBD domain of SARS-CoV-2 (upper line) and SARS-CoV (bottom line) aligned using BLASTP. Amino acid residues involved in the interaction (designated by capital bold letters) between FGFR3 (marked with a *) and ACE2 (marked with a ^) were identified using the YASARA Structure and literature data [97]. Promiscuous AA of the SARS-CoV-2 RBD involved in binding both FGFR3 and ACE2 are marked with *^. Members of AA cluster D442, N448, Y449, S494, Q493, L492, corresponding to phage mimotope EDYSELVSQ, are underlined and marked with (+).

To determine the mechanism of virus selectivity towards canonical and alternative receptors, we compared AA clusters and the related phage mimotopes responsible for binding to ACE2 and FGFR3 (Figure 12). It was found that although binding of the spike RBD to FGFR3 is much weaker than binding to ACE2 and CD26 (Dipeptidyl peptidease-4 or DPP4), in a direct ELISA, the presence of amino acids R403, D405, K444, E484, and N501 that determine the interaction of S protein of SARS-CoV-2 with FGFR3, which are absent in preceding coronavirus variant SARS-CoV, may be advantageous for virus infectivity.

These observation are consistent with the hypothesis that the evolution of the virus in the human population can occur not only by escaping immune pressure or increasing the affinity of RBD towards the canonical ACE2 receptor, as commonly assumed [98], but also through adaptation of the virus to alternative receptors and co-receptors of expressed on-host cells. In the retrospective analysis, the discovered cluster of FGFR interacting with SARS-CoV-2 spike amino acids was compared with corresponding amino acids of the SARS-CoV spike. Intriguingly, all of these amino acids mutated to the potential FGFR-binding cluster (R403K, E406D, N439R, K444T, V483P, E484A, and N501T), while members of the AA cluster (D442D, N448N, Y449Y, S494D, Q493N, and L492l) corresponding to phage mimotope EDYSELVSQ were mostly equally presented on both the SARS-CoV-2 and SARS-CoV RBD domains. Considering that both the virus and phage could emerge during their interaction with human growth factor receptors overexpressed on the host Calu-3 cells, one can speculate that the SARS-CoV-2 virus emerged during adaptation of SARS-CoV to Calu-3 or other human cancer cells. These observations are consistent with the hypothesis that the evolution of the virus can occur both by escaping immune pressure in the human population, and through the exchange of viral receptors and co-receptors during virus adaptation to host cells.

## 4. Discussion

### 4.1. Key Role of Human Protein Receptors in SARS-CoV-2 Infection and Evolution

The evolution of human coronaviruses during recent decades can be followed by comparing seven variants, starting from HCoV-229E (1962), and continuing to the last outbreak of SARS-CoV-2 (2019) [4] and its mutated variants. In particular, the emergence of the highly pathogenic coronaviruses SARS-CoV and MERS-CoV [29] inspired significant activities in uncovering the molecular mechanisms of viral infectivity and pathogenesis [33,37,99,100,101]. The spike S protein (Figure 1) was identified as a mediator of virus and host cell binding [102], while human angiotensin-converting enzyme 2 (hACE2) was recognized as a receptor of SARS-CoV and SARS-CoV-2. Furthermore, several other receptors and cellular proteases were found to support viral entry in the host cells [1,101]. Intriguingly, the spike RBD and appropriate human protein receptors diverge among human coronaviruses [37]. For example, ACE2 serves as a receptor for NL63, SARS1, and SARS2 strains; aminopeptidase N (CD13) serves as a receptor for the 229E strain; dipeptidyl peptidase-4 (DPP4 or CD26) serves as a receptor for MERS [29]; while peptidases ANPEP, ENPEP, and AGTR2 are related to SARS-CoV-2 infectivity [37].

### 4.2. Alternative Receptors of Coronaviruses as an “Operational Reserve” for the Evolutionary Escape of Emerging SARS-CoV-2 Variants

Despite minor changes in the spike conformation of human coronaviruses, single mutations in their S proteins can dramatically change the pathogenicity [99]. For example, the enhanced infectivity of SARS-CoV-2 can be attributed to the use of alternative receptors by the virus during the evolution of its predecessors [37]. Thus, the lower infectivity but higher mortality rates of SARS-CoV—the source of the epidemic in 2003 [40]—in comparison with SARS-CoV-2, can be explained in part by the appearance of the mutation K403R in the spike protein of the SARS-CoV-2 variant [84] (Figure 12). This mutation resulted in the creation of an integrin-binding motif, RGD, which is crucial for a successful infection that is known to drive the infectivity of a broad spectrum of viruses [103]. The presence of the RGD site was also thought to be a factor that can boost the affinity of SARS-CoV-2 to ACE2-positive target cell, and ACE2-negative cells. Thus, the RGD motif in the spike protein of SARS-CoV-2 could be critical in infecting cells through the RGD-binding integrins [36]. In their turn, RGD-binding integrins are involved in the activation of transforming growth factor beta (TGF-β), which can be related to complications in COVID-19 patients [36]. This is a good illustration of how a single mutation might be able to redirect a virus to a reserve receptor and assist in the dramatic increase of viral spreading and pathogenicity. A number of other non-canonical receptors were proposed as potential entry points of the virus, such as basigin (CD147) or the tyrosine-protein kinase receptor UFO (AXL) receptor that can mediate the invasion of the SARS-CoV-2 virus into host cells [104,105]. The revealed importance of CD147 in virus infection inspired a search for other potential receptors of the SARS-CoV-2 virus using computational methods [106]. A representative member of the GFR family—fibroblast growth factor receptors (FGFRs)—are TKRs that play an important role in cell proliferation, migration, differentiation, and carcinogenesis [80], and are also found to be relevant in viral infections, for example, during endocytosis of the adeno-associated virus 2, and can be considered as a reserve receptor for SARS-CoV-2.

### 4.3. Development of Phage-Derived Probes for Monitoring Virus Evolution

Considering the different mechanisms of SARS-CoV-2 infectivity and evolution, including the use of alternative receptors, the knowledge and monitoring of receptor specificity for emerging variants is important in the creation of new antiviral vaccines and medicines. Currently used methods of structural and biochemical analysis, including computer analysis, are not well suited to meet the challenging problem of global screening and monitoring the origins of emerging diseases, such as COVID-19 [106]. Considering the importance of the problem and the lack of simple and reliable approaches to its solution, we thought about phage probes that could compete with pseudoviruses prepared from emerged virus variants in cell culture or animal models and produce a first warning signal regarding a change in virus behavior. In opposition to spike-binding antibodies commonly used to study the interaction of the ACE2 receptor with SARS-CoV-2 variants in vitro, phage spike mimotopes targeted to other cellular receptors would allow the discovery of their role in viral infection in vivo using cell culture, tissue samples, organs, or the whole organism. Phage probes, and specifically landscape phage probes, previously demonstrated several remarkable features, which determined their use in detection of biological threats [44,53,82,107,108,109,110,111,112,113]. The phage mimicry approach, previously suggested [31] for epitope discovery, was essentially modified and adapted for receptor binder discovery.

We evaluated the performance of the phage mimicry approach in the preparation of phage-derived probes targeted to a representative of the GFR family—fibroblast growth factor receptors (FGFR) [80]. Following the phage mimicry strategy, we assumed that if selected cell-associated phage mimotopes are related, for example, to FGF1 or NRP1 (the natural partners of FGFR3 [81,85,86,87,88]), then they can be related to the mimotopes of the spike S protein, which also bind to FGFR3. To realize this idea, we focused on NRP1, which was shown to form a complex with FGFR3. Since the 3D structure of the complex is unknown, we used methods of molecular modelling. In the similar way, we determined a structure of FGFR3 with the SARS-CoV-2 spike RBD and NRP1-FGFR3. We found that the binding AA clusters of NRP1 have a striking similarity to FGF1 and the RBD, as shown in Figure 8 and Table 2. An ELISA was used to confirm our predictions. In this study, a panel of closely related phage mimotopes mimicking the receptor-binding sites on the surface of FGF1, NRP1, and the SARS-CoV-2 spike protein have been identified, and their binding to FGFR3 was shown by an ELISA and confirmed by molecular modelling. Remarkably, it was observed that some of the amino acids of phage mimotopes, and the corresponding spike RBD AA clusters interacting with FGFR3, were absent in the RBD of the SARS-CoV variant [40] (Figure 12). We hypothesize that these residues have been mutated in the emerged variants of the SARS-CoV-2 virus and have granted them a more aggressive phenotype [4]. In relation to these observations, it is interesting to note that phage EDYSELVSQ has been isolated by affinity selection from Calu-3 cells—a representative cell line of non-small cell lung cancer that overexpress human growth factor receptors and other important cellular receptors. Calu-3 cells are sensitive to SARS-CoV-2 infection and, like other human cancer cells, are commonly used for propagation of the SARS-CoV and SARS-CoV-2 viruses in cell culture [30,72,77,114,115,116,117]. These observation are consistent with the hypothesis that the evolution of the virus in the human population can occur not only by escaping immune pressure and increasing the affinity of RBD to ACE2, as commonly assumed [98], but also through exchanging virus receptors and co-receptors during the adaptation to a new host cell [1,10].

## 5. Conclusions

In this study, a panel of phage mimotopes, closely related to the FGF1, NRP1, and spike receptor-binding AA clusters, were identified. Their binding to ACE2 and FGFR3 was shown by an ELISA and confirmed by molecular modelling. The phage probes that compete with viruses for binding to certain cellular receptors can provide essential information about the mechanisms of virus infectivity. They can also serve as leads in the development of vaccines or drugs, or be used as interfaces in diagnostics. Here, we demonstrated the phage mimicry strategy and developed experimental algorithms that are being used in our ongoing project to evaluate the role of DPP4, NRP1, EGFR, and other human receptors that may be involved in SARS-CoV-2 evolution. We note that this in vitro system is very limited and further studies are required, using more complete pseudoviral and cell receptor expression systems. These additional studies in more advanced systems are beyond the scope of this proof-of-concept project but have led us to identify potential candidate mimotopes for further study.

## Figures and Tables

**Table 1:** Characteristics of recombinant human cell receptors and viral spike proteins.

Protein	Vendor	Catalog #	Residues		Length	Molecular Weight (kDa)
			Start	End		
hACE2	Sino Biological	10108-H08H	Q18	S740	734	85.1
hDPP4	Sino Biological	10688-H08H	D34	P766	744	86.3
hNRP1	Sino Biological	10011-H08H	F22	K644	634	71.3
hFGFR3	Sino Biological	16044-H08H	E23	G375	364	39.6
SARS-CoV-2 S1 spike	Sino Biological	40591-V08H	V16	R685	681	76.5

**Table 2:** Amino acid clusters identified on the surface of ligands and their corresponding phage mimotopes. Bold letters highlight the amino acid residues shared with the phage mimotope.

Phage Mimotope	Amino Acid Clusters Identified in:		
	FGF1	NRP1	Spike RBD
AGSGQEYLD	T**G**-**GQ**S**Y**--	**A**S**SG**-**E**--**D**	**AGS**-**Q**-**Y**I-
DGRADLSYD	**DGR**-**D**-**S**-**D**	**D**S**R**G**EL**N**YE**	-**G**NS**DLSY**N
DGRMTVYNE	**DGR**L**TV**-G**D**	**DGR**-NM-**NE**	**DGR**--**VYN**Q
DGRRDVADD	**DG**-**RD**-S**D**T	**EG**-**RD**FGN**D**	**DG**Q**REV**G**D**-
DSFVNAPED	**DT**-**V**DL**P**H**D**	**D**-**F**-**N**G-**ED**	-A**FVN**G**PE**-
EDYSELVSQ	**E**NH-**EL**---	**EDFSE**FT-**Q**	**D**N**YS**Q**LVS**-
ETRVEPEYD	**ET**Q**V**-**P**K**Y**N	--**R**-**DPEYD**	**E**V**R**AQ**P**-**Y**-
VNADSASSD	**V**DR**DS**R**SS**-	**V**P**A**K**S**T**SSD**	L**N**-**DS**V**SS**-
VPTDTDYSD	-**P**-**DTDYS**-	**VP**—**STDYS**	**VP**N**DS**N**YS**-
VQAFDDSVD	T**Q**S**Y**G**DTVD**	T**Q**-**FD**Q**S**-**D**	I**QAY**-**ES**I**D**
VTALEPGQD	I-**ALE**-**G**E-	-**SA**I**EPGQD**	-**TA**V**EPG**--

## Data Availability

Data is contained within the article or are available from publicly available datasets. Any additional data is available on request from the corresponding author.
